# Development and Verification of an Adjustment Factor for Determining the Axial Length Using Optical Biometry in Silicone Oil-Filled Eyes

**DOI:** 10.3390/diagnostics12010163

**Published:** 2022-01-11

**Authors:** Gerd U. Auffarth, Tadas Naujokaitis, Louise Blöck, Anna Daghbashyan, Jan Meis, Victor A. Augustin, Ramin Khoramnia, Timur M. Yildirim

**Affiliations:** 1 International Vision Correction Research Centre, Department of Ophthalmology, University of Heidelberg, 69120 Heidelberg, Germany; Tadas.Naujokaitis@med.uni-heidelberg.de (T.N.); Louise.Bloeck@med.uni-heidelberg.de (L.B.); Anna.Daghbashyan@med.uni-heidelberg.de (A.D.); victor.augustin@med.uni-heidelberg.de (V.A.A.); Ramin.Khoramnia@med.uni-heidelberg.de (R.K.); timur.yildirim@med.uni-heidelberg.de (T.M.Y.); 2 Institute of Medical Biometry, University of Heidelberg, 69120 Heidelberg, Germany; meis@imbi.uni-heidelberg.de

**Keywords:** ocular biometry, optical coherence tomography, partial coherence interferometry, Scheimpflug imaging, retinal disease, refractive outcome, optical diagnosis, treatment options

## Abstract

The aim of this prospective clinical study was to establish and verify an adaptation for axial length (AL) measurement in silicone oil (SO)-filled pseudophakic eyes with a Scheimpflug and partial coherence interferometry (PCI)-based biometer. The AL was measured with a Pentacam AXL (OCULUS Optikgeräte GmbH, Wetzler, Germany) and IOLMaster 700 (Carl Zeiss Meditec, Jena, Germany). The coefficients of variation (CoV) and the mean systematic difference (95% confidence interval (CI)) between the devices were calculated. After implementing a setting for measuring AL in tamponaded eyes with a Pentacam based on data of 29 eyes, another 12 eyes were examined for verification. The mean AL obtained with the Pentacam was 25.53 ± 1.94 mm (range: 21.70 to 30.76 mm), and with IOLMaster, 24.73 ± 1.97 mm (ranged 20.84 to 29.92 mm), resulting in a mean offset of 0.80 ± 0.08 mm (95% CI: 0.77, 0.83 mm), *p* < 0.001. The AL values of both devices showed a strong linear correlation (r = 0.999). Verification data confirmed good agreement, with a statistically and clinically non-significant mean difference of 0.02 ± 0.04 (95% CI: −0.01, 0.05) mm, *p* = 0.134. We implemented a specific adaptation for obtaining reliable AL values in SO-filled eyes with the Pentacam AXL.

## 1. Introduction

Accurate measurement of the axial length (AL) is essential for any ocular intervention that changes the lens’s geometry, position, or its composition. In phakic and pseudophakic eyes, modern ocular biometers allow one to obtain precise AL values to predict the eye’s postoperative refraction reliably. In the beginning of intraocular lens (IOL) surgery, manual ultrasound A-scans were used to measure AL. Apart from handling difficulties, this method is limited by a longitudinal resolution of 200 μm and an accuracy of 100–120 μm, which translates in a postoperative refractive error of 0.28 D per 100 µm [1,2]. Early automated ocular biometers were based on optical A-scan technology. The first device based on partial coherence interferometry (PCI), the IOLMaster (Carl Zeiss, Jena, Germany), was introduced in 1999, and has been subsequently adopted and improved [3]. The most recent generation of the IOLMaster (IOLMaster 700) uses swept-source optical coherence tomography (SS-OCT) to determine the ocular AL [4]. The device uses a vertical-cavity surface-emitting laser with a wavelength of 1055 nm to obtain scans with a width of 6 mm and a measurement speed of 2000 A-scans per second. This allows for a scan depth of 44 mm with a resolution of 22 µm [5,6]. The Pentacam (Oculus, Wetzlar, Germany) was introduced in 2002 to provide high-resolution biometry data of the anterior segment using Scheimpflug technology. The device has been shown to provide superior reproducibility in measuring anterior segment geometry compared to other technologies [7]. As a comprehensive ocular biometer not only requires precise measurements in the anterior ocular segment, but also the axial length, the Pentacam AXL was introduced in 2015 to provide contact-free optical biometry from the corneal surface to the retinal pigment epithelium (RPE) based on a combination of Scheimpflug and PCI technologies. For AL measurement, the device uses an infra-red light-source with a wavelength of 880 nm. The measurement is based on six pulses per examination, with an individual pulse duration of 520 ms [8].

However, axial length measurement depends not only on the optical biometer’s technology, but also on the refractive properties of the media in the path of light travelling from an object to the RPE. In functional terms, the optical biometer’s accuracy and repeatability are determined mainly by the spectral calibration in swept-source and spectrometer-based optical biometers. An effect of spectral calibration could potentially cause the differences between the biometers’ measurements [9,10]. In some disease treatments, the natural vitreous is exchanged for media that have different optical properties. In cases of severe retinal pathologies, an endotamponade of silicone oil (SO) is placed in the vitreous cavity after pars plana vitrectomy (PPV) to stabilize the retina [11]. Such a tamponade considerably influences the precision of the AL measurement, as it can attenuate the light energy reaching the RPE [12]. Therefore, the device settings for regular AL measurement require to be adjusted with a compensatory systematic offset. In conducting ocular biometry measurements on endotamponaded patients, for example, in preparation for a surgical intervention after an IOL luxation, preoperative refinement can contribute to improving the patient’s subjective quality of vision. This could lead to the avoidance of further surgery.

Therefore, the aim of this study was to establish and verify an adaptation for determining the AL using the Pentacam AXL in eyes that have been treated with SO tamponade. Additionally, the repeatability and agreement of AL measurements in SO-filled eyes were assessed with two optical biometers, the IOLMaster 700 and the Pentacam AXL.

## 2. Materials and Methods

### 2.1. Study Design and Patient Recruitment

In this prospective, single-center, clinical study, the ALs of pseudophakic SO-filled eyes were measured with two optical biometers in a random order, the Pentacam AXL (OCULUS Optikgeräte GmbH, Wetzler, Germany) and the IOLMaster 700 (Carl Zeiss Meditec, Jena, Germany). Thirty eyes of thirty consecutive patients were included between September 2018 and January 2020. All patients had previously undergone pars plana vitrectomy with implantation of a polydimethylsiloxane endotemponade—either Siluron 5000 (Fluron GmbH, Ulm, Germany) in 28 cases, or Vitreo Sil 5000 (Oculentis BV, Berlin, Germany) in 2 cases, for the following ocular diseases: rhegmatogenous retinal detachment in 24 cases, tractional retinal detachment in diabetic retinopathy in 2 cases, and 1 case each of endophthalmitis, macular hole, epiretinal membrane, and hypotony. There were no exclusion criteria in terms of ocular pathologies.

### 2.2. Repeatability and Agreement

Three consecutive AL measurements were obtained with IOLMaster using the built-in setting for SO-filled pseudophakic eyes; and with Pentacam using the setting for pseudophakic eyes. AL measurements that did not fulfill the quality requirements suggested by the device-manufacturers (QS = OK for Pentacam, no exclamation mark for IOLMaster) were excluded from our data analysis. By pooling the within-subject variances, the repeated measurements were used to calculate coefficients of variation (CoV) to judge the repeatability of measurements for each device. Measurements were then averaged on a subject-level basis, and means (±standard deviations, SD) were calculated for both devices. Agreement between devices was illustrated using a Bland–Altman analysis. The mean offset (95% confidence interval (CI)) between both devices was calculated.

### 2.3. Verification

After implementing the new setting for measuring the AL in SO-filled pseudophakic eyes with *p* by adjusting for the mean offset, a verification cohort was enrolled prospectively. Another twelve consecutively recruited patients were chosen using the same inclusion criteria as described in 2.2. Assuming a standard deviation of 0.08, a paired *t*-test with a significance level of 0.05 would have a power of 88% to detect a difference of 0.08 mm in twelve paired samples.

### 2.4. Data Analysis

Statistical analysis was performed using SPSS for Windows software (version 26; IBM Corporation), and Microsoft Excel 2013. Power calculations were performed using PASS version 16.0.12. Normality of AL data was tested using the Shapiro–Wilk test. A two-sided *t*-test for paired samples was used to test AL differences between both devices. A *p* value less than 0.05 was considered statistically significant.

## 3. Results

**Figure 1:**
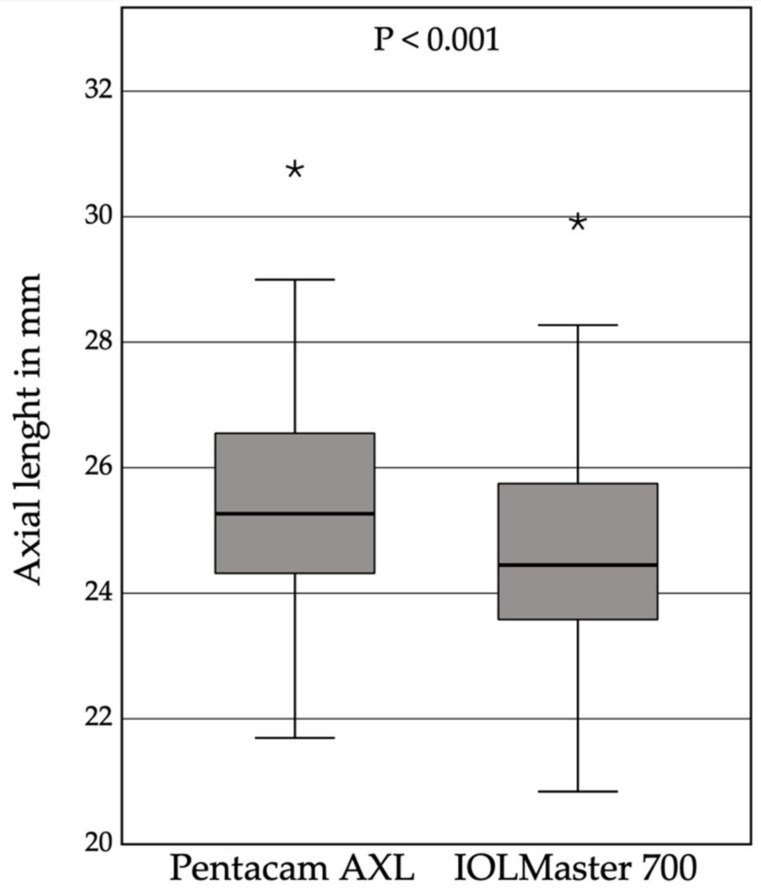
The boxplots of the mean axial length results of three consecutive measurements with the Pentacam AXL using the pseudophakic setting (25.53 ±1.94 mm), and the IOLMaster 700 using the silicone oil pseudophakic setting (24.73 ± 1.97 mm), reveals a mean systematic offset of 0.80 ±0.08 mm, (95% CI: 0.77, 0.83 mm), * outliers, *p* < 0.001.

In 29 out of 30 SO-filled eyes, 3 consecutive measurements could be obtained with each device. One eye was excluded from the study because patient incompliance prevented completion of all six consecutive measurements. Thus, the offset cohort consisted of 29 eyes (15 right eyes) of 29 patients (23 male), with a mean age of 64 ± 9.5 years. Mean ALs obtained with the Pentacam were 25.53 ± 1.94 mm (range: 21.70 to 30.76 mm), and with IOLMaster, 24.73 ± 1.97 mm (range: 20.84 to 29.92 mm) (Figure 1).

**Figure 2:**
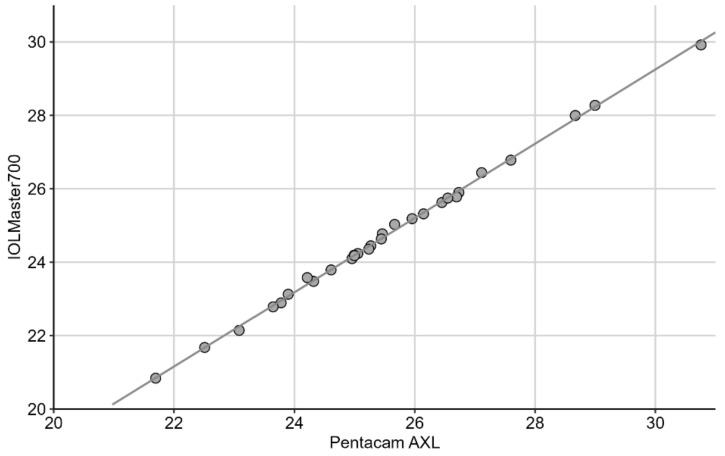
The linear regression model demonstrating a very strong correlation between the axial lengths measured with the Pentacam and the IOLMaster (r = 0.999).

The mean systematic difference between both devices was 0.80 ± 0.08 mm (95% CI: 0.77, 0.83 mm), *p* < 0.001. For both devices, the CoV was below 1% (0.26 and 0.09 %, for *p* and I, respectively). The AL data showed a very strong linear correlation (r = 0.999) (Figure 2).

**Figure 3:**
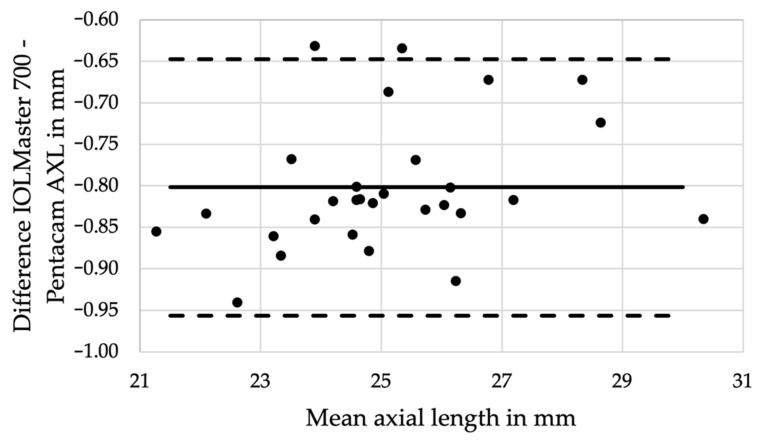
The Bland–Altman analysis confirms good agreement of the axial length measurements between the two devices independent from the axial length with an offset of 0.80 ±0.08 mm (95% CI: 0.77–0.83 mm), *p* < 0.001.

The Bland-Altman analysis showed that data was independent of the axial length considering the offset, and distributed close to the mean difference, suggesting good agreement between both devices (Figure 3).

The verification cohort confirmed the agreement between the two study devices, with a statistically and clinically non-significant mean difference of 0.02 ± 0.04 (95% CI: −0.01, 0.05) mm, *p* = 0.134 (Table 1).

## 4. Discussion

This prospective clinical study allowed us to implement and verify a specific setting for determining the AL in eyes filled with SO with the Pentacam AXL accurately and repeatably.

Eyes that previously underwent PPV with SO implantation usually suffer from severe ocular diseases, and this is also seen in the demographics of the current study cohort. Refinement of preoperative measurements is especially important in such eyes to create the best starting conditions for any additional surgery. Thus, implementing specific measurement adoptions for special patient subgroups broadens the application spectrum of ocular biometers, and can provide a more personalized approach, which is especially valuable in the management of severely diseased eyes.

When calculating the AL of an eye, the refractive index of each ocular medium must be considered. In healthy eyes, the most common approach is to use an equivalent refractive index for a whole intraocular passage, which can be either adopted to specific formulas, or implemented in the ocular biometer. Each index is optimized for a specific ocular configuration. The PCI-based IOLMaster biometers incorporate an equivalent refractive index of about 1.3549, and are optimized for an AL of 24 mm with a lens thickness of 3.6 mm [13]. In any other configuration, an optimization for the specific subgroups, e.g., with different refractive indices, such as patients with an SO tamponade, needs to be applied. However, even after adjusting for differences in the refractive index, measuring the AL in SO-filled eyes carries additional challenges: artifacts due to oil droplets may complicate the measurement, and varying degrees of SO-underfill might bias the result, especially in ultrasound biometry with the patient in a prostrate position [14].

The coefficient of variance (CoV) can be used to express the precision and repeatability of a measurement, where lower CoV values indicate higher accuracy. This metric has previously been used to report the quality of a measurements obtained with different biometers, including the IOLMaster and Pentacam [15,16,17,18]. In 2017, Ruiz-Mesa et al. compared a Scheimpflug/PCI-based system with an optical low-coherence reflectometry (OLCR) device. Authors reported a CoV of 0.65 and 0.16% for measuring the AL with the Scheimpflug/PCI-based and the OLCR device, respectively [18]. The latest biometers yield even better results: in a prospective clinical trial presented in 2021, Fişuş et al. included 50 routine cataract patients to compare the repeatability of two SS-OCT biometers, the IOLMaster 700 and the Anterion (Heidelberg Engineering GmbH, Heidelberg, Germany), and one OLCR device, the Lenstar LS 900 (Haag-Streit AG, Köniz, Switzerland). For AL values, the CoV ranged between 0.006 and 0.012% [17]. In another recent study by Shetty et al. comparing the Lenstar LS 900, IOLMaster 700, and Anterion, the CoVs for AL ranged between 0.029 and 0.058% [16].

The data we present showed slightly higher CoVs of 0.26 and 0.09% for the Pentacam AXL and the IOLMaster 700, respectively (0.11 and 0.03 % in the verification group). However, our study was not performed in healthy eyes, and only included severely diseased eyes with SO filling, which complicates the measurement. Therefore, with CoVs well below 1%, our study suggests that both devices still offer a high repeatability despite more difficult conditions [12].

A few former studies also compared the accuracy and reliability of AL measurement in SO-filled eyes with different methods. Kunavisarut et al. performed a prospective clinical study of the IOLMaster 500 versus A-scan immersion biometry. The authors measured the AL in thirty-four SO-filled phakic eyes before and three months after SO removal and cataract surgery using both methods. Additionally, they obtained the manifest refraction to verify their results. The IOLMaster provided more accurate values with less deviation of the predictive postoperative refractive error than the A-scan immersion method [19]. This finding is in accordance with the results of our study, which also showed a good repeatability. As optical AL measurement might even depend on the specific type of SO, Roessler et al. compared the effect of different SOs on the AL measurement. In their study, the authors compared the mean AL of 26 eyes that were either filled with conventional or heavy SO with 16 contralateral eyes. The authors reported acceptable values for accuracy and signal quality in the SO group, which was similar in both SO-subgroups, but worse compared to the contralateral healthy controls. Even though the accuracy in healthy eyes was higher, the study showed that PCI provided good accuracy and signal quality for AL measurement in both types of SO [20]. Most of the eyes in our study were treated with a single type of SO (93% Siluron 5000), resulting in a homogenous patient cohort, and minimizing a bias due to different material properties.

One limitation of our study is that we take the “true AL” to be the one provided by the IOLMaster 700 using its built-in SO pseudophakic setting. We could have taken a different approach. We could have used another value as the “true AL”, e.g., the measured length before endotamponade implantation, or one taken after its removal. However, implantation of a tamponade might itself lead to changes of the AL, as suggested by Liu and Li, who compared different biometry data before and after SO implantation in 63 patients [21]. Thus, we decided to use the most recent and most researched ocular biometer, the IOLMaster, which has been proven to provide excellent reliability for AL measurements, as shown in different former studies as a reference device [14,19,20,21,22].

Regarding the quality of AL measurements in this more difficult patient cohort, our study showed that the Pentacam AXL yielded a high level of accuracy and repeatability with similarly low SD and CoV compared to the IOLMaster 700. Furthermore, to the best of our knowledge, the presented study is the first to report on AL data of the Pentacam AXL in SO-filled eyes.

## 5. Conclusions

We implemented a specific adaptation setting in the Pentacam AXL in order to obtain accurate AL values in SO-filled pseudophakic eyes. The Pentacam AXL and the IOLMaster 700 showed a high degree of agreement, and both devices provided excellent repeatability. Results of our study were used by the manufacturer to adjust the device software, and provide a new feature that is specifically for use in measuring pseudophakic SO-filled eyes.

## Figures and Tables

**Table 1:** Axial length measurements of the verification group using two optical biometers.

	IOLMaster 700	Pentacam AXL	Paired Differences
mean [mm] ± SD	24.22 ± 2.06	24.24 ± 2.04	0.02 ± 0.04
95% CI	22.91, 25.53	22.95, 25.53	−0.01, 0.05
min, max	20.43, 28.47	20.45, 28.37	−0.10, 0.06
CoV [%]	0.03	0.11	

CI, confidence interval for the mean estimate; CoV, coefficients of variation.

## Data Availability

All data generated or analyzed during this study are included in this published article.
